# *In vitro* Digestibility of Dietary Carbohydrates: Toward a Standardized Methodology Beyond Amylolytic and Microbial Enzymes

**DOI:** 10.3389/fnut.2019.00061

**Published:** 2019-05-07

**Authors:** Oswaldo Hernandez-Hernandez, Agustín Olano, Robert A. Rastall, F. Javier Moreno

**Affiliations:** ^1^Instituto de Investigación en Ciencias de la Alimentación, CIAL (CSIC-UAM), Campus de la Universidad Autónoma de Madrid, Madrid, Spain; ^2^Department of Food and Nutritional Sciences, The University of Reading, Reading, United Kingdom

**Keywords:** non-digestible carbohydrates, slowly digestible carbohydrates, brush border enzymes, carbohydrases, non-starch oligosaccharides, small intestinal brush border membrane vesicles

## Background and Limitations of the Conventional *in vitro* Methods to Assess Digestibility of Dietary Carbohydrates

Scientific evidence of human digestion of foods gathered during the last decades has strengthened the relationship between digestibility and the possible effects of foods on human health (1). The high complexity of the gastrointestinal (GI) environment and the lack of an easy direct access to most of the parts of the GI tract (2) with the exception of the oral cavity, prevent the broad implementation of *in vivo* animal, or human trials for assessing the digestion, absorption, and metabolism of dietary food ingredients. Therefore, *in vitro* testing has a central place in the current investigations of food's digestive fate.

Non- or slowly digestible carbohydrates may have appealing properties that are associated with a series of beneficial physiological effects, such as low-calorie (important in preventing obesity), low-glycemic (helpful in managing diabetes and cardiovascular disease), and low-digestible (helpful in reducing the intestinal transit time and in positively modulating the gut microbiota composition and activity). These beneficial effects have sparked the interest, from both academic and industry perspectives, in carbohydrates showing resistance to digestion (or slow digestibility) and absorption in the small intestine and thus being available in the large intestine as substrates for fermentation by gut microbiota.

Since Southgate published, in the late 1960s, two methods for measuring available (3) and unavailable (4) carbohydrates in foods by hydrolyzing starch with amyloglucosidase and pullulanase of fungal origin, a plethora of models ensued with the common rationale of using amylolytic enzymes that, in some cases, may be combined with a microbial invertase (5). Remarkably, the substrate specificity, hydrolytic mechanism and influence in physiologic responses of amylolytic enzymes from fungal or bacterial sources commonly used in *in vitro* starch digestion assays has been demonstrated to be noticeably different from their equivalent in mammals (6, 7).

The current state-of-the-art reveals that human gastrointestinal digestion of dietary carbohydrates is a multistage process, involving up to six different carbohydrases produced by three different organs (Table 1). The main function of the oral phase is the conversion of food into a homogenous mass during mastication, whilst the starchy carbohydrates undergo a very limited salivary α-amylolysis. The main site for carbohydrate digestion is the small intestine, where the chyme is mixed with α-amylase containing secretions of the pancreas. In addition, the key role played by the mucosal disaccharidases embedded in the small intestinal brush border membrane vesicles that are responsible for the final stage of luminal digestion prior to absorption needs to be recognized (11, 12) (Table 1). Strikingly, the inclusion of small intestine mucosal carbohydrases has been ignored in the vast majority of *in vitro* models developed to date. This issue is particularly critical in the assessment of not only non-starchy carbohydrates, but also in the quality of starch digestion since the small intestine mucosal α-glucosidases have been reported to play an important role by influencing the rate, location and extent of glucose release which is associated with high glycaemia-related health issues such as diabetes and other metabolic syndromes (7). Nevertheless, recent research is addressing this caveat by developing alternative *in vitro* methodology aiming to better model the interaction of the carbohydrate with small intestine mucosal carbohydrases as explained below.

**Table 1:** Human carbohydrases involved in dietary carbohydrate digestion.

**Digestive carbohydrases**	**Type of enzyme**	**Glycoside hydrolase family^a^**	**Production organ/main site of digestion**	**Glycosidic linkage specificity**	**Main substrates^b^**	**Main products^b^**
Salivary α-amylase^c^	Secreted (α-glucosidase)	13	Salivary gland/mouth	Glcα(1→ 4)Glc	Starch; linear maltooligosaccharides (*n* > 6)	Maltose; maltotriose; α-dextrins
Pancreatic α-amylase^c^	Secreted (α-glucosidase)	13	Pancreas/Small intestine	Glcα(1→ 4)Glc	Starch; linear maltooligosaccharides (*n* > 6)	Maltose; maltotriose; α-dextrins
Sucrase-isomaltase	Mucosal (α-glucosidase)	31	Small intestine (brush border membrane)/Small intestine	Glcα(1⇔2)βFru Glcα(1→ 4)Glc Glcα(1→ 6)Glc	Sucrose; isomaltose; maltose; maltotriose; α-dextrins	Glucose; fructose
Maltase-glucoamylase	Mucosal (α-glucosidase)	31	Small intestine (brush border membrane)/Small intestine	Glcα(1→ 4)Glc Glcα(1→ 6)Glc	Linear and branched maltooligosaccharides (*n* = 2–9)	Glucose
Lactase-phlorizin hydrolase	Mucosal (β-glycosidase)	1	Small intestine (brush border membrane)/Small intestine	Glcβ(1→ 4)Gal Glcβ(1→ 4)Glc	Lactose, cellobiose, cellotriose, cellulose	Glucose; galactose
Trehalase	Mucosal (α-glucosidase)	37	Small intestine (brush border membrane)/Small intestine	Glcα(1⇔1)αGlc	Trehalose	Glucose

a *According to CAZy database (http://www.cazy.org/) (8)*.

b *Based on and updated from Alpers (9)*.

c *Human salivary and pancreatic α-amylases have 94% amino acid identity although they are encoded by different genes (10)*.

## Development of Alternative *in vitro* Models to Assess Carbohydrate Digestibility Using Mammalian Small Intestinal Brush Border Carbohydrases

### TIMcarbo: A Dynamic GI Tract Model Mimicking the *in vivo* Physical Processing and Temporal Changes in Luminal Conditions

This method has been built on the well-known TNO Gastro-Intestinal Model (TIM), which is a multi-compartmental dynamic model that was developed by TNO in The Netherlands in the early 1990s in response to industrial demand to study food products under more physiologically relevant conditions as compared to contemporary digestion models (13, 14). The TIMcarbo model includes an oral digestion phase by using a model of artificial mastication with a bacterial salivary α-amylase. After the luminal gastrointestinal digestion, which is simulated by adding pancreatic juice containing proteases, lipases and amylase, among other factors, the TIMcarbo mimics the carbohydrate uptake by the epithelium. This is achieved by dialysis of the products of digestion by a commercial rat brush border enzyme extract complemented with a bacterial β-galactosidase (15). The use of a microbial lactase is justified by the fact that the rat brush border extract contains a much lower activity than that found in humans. Finally, the *in vitro* methodology is combined with an *in silico* kinetic modeling that allows the prediction of glycemic response curves in humans.

This model is highly relevant to studying the digestion of whole foods and meals where the physical condition of the digesta changes over time, e.g., viscosity or particle size reduction that are key determinants of the rate of carbohydrate digestion and absorption *in vivo* (5, 16, 17). However, this refined method is not so suitable for screening studies or for testing the digestibility of carbohydrates available at low amounts, which is usually a limiting factor when the digestibility of novel carbohydrates produced at laboratory scale needs to be assessed.

### Batch *in vitro* Digestion Assays Using Rat or Pig Small Intestine Mucosal Carbohydrases

Batch models do not pretend to mimic the physical and mechanical processes that occur *in vivo*. Consequently, in this type of digestion model the products of digestion are not removed and therefore, they do not model the absorption process. However, this type of model is especially suited for assessing the digestibility of isolated carbohydrates or single foods and can provide valuable information on mechanistic studies.

In line with the brush border enzyme digestion included in the TIMCarbo model, the use of rat small intestinal extract has been successfully applied for the assessment of digestibility of several prebiotic carbohydrates. These are non-digestible carbohydrates with the ability to selectively modulate the composition and/or activity of the gut microbiota, thus conferring benefit(s) upon host health (18), such as fructooligosaccharides (19), galactooligosaccharides (20, 21), or isomaltooligosaccharides (22).

In addition to the use of the rat intestinal extract, Lee et al. (23) investigated the contribution of the four main individual recombinant mucosal α-glucosidases (that is, glucoamylase, maltase, sucrase and isomaltase) on a range of unusual α-linked glycemic disaccharides with two glucose units. These authors provided intriguing differences in hydrolytic properties of the individual rat mucosal α-glucosidases to disaccharide substrates differing in glycosidic linkages. Furthermore, they concluded that mammalian mucosal carbohydrases must be used in *in vitro* assessment of digestion of glycemic carbohydrates instead of microbial digestive enzymes. More recently, a newly developed *in vitro* digestion model, based on the use of a commercial rat small intestinal extract under physiological conditions of temperature and pH, was successful for evaluating the digestibility of dietary oligosaccharides of degree of polymerization up to four with a requirement for a minimum of 0.5 mg of carbohydrate (24). This method demonstrated its capacity to distinguish between well-recognized digestible and non-digestible carbohydrates; the tested digestible carbohydrates were readily hydrolyzed whereas the oligosaccharides classified as non-digestible were barely hydrolyzed. Remarkably, a good correlation was found between this *in vitro* method and *in vivo* data collected on growing (25) or neonatal (26) rats and from aspiration of the gut content at the terminal ileum of healthy humans (27) on galactooligosaccharides and fructooligosaccharides digestion.

The high physiological and anatomical similarity of the pig and human digestive tracts (28) may provide additional advantages of using pig small intestinal material instead of that from rats. Thus, alternative methods based on the use of mammalian intestinal enzymes derived from pigs (6) or weaning piglets (29) have recently been proposed. Moreover, Tanabe et al. (30) successfully applied an improved AOAC 2009.01 method by using porcine small intestinal enzyme instead of fungal amyloglucosidase to accurately determine non-digestible oligosaccharides in marketed food products. In this context, the use of small intestinal brush border membrane vesicles conveniently isolated and purified from post-weaned pigs has recently shown to be a reliable and straightforward strategy to evaluate prebiotic carbohydrate digestibility, under physiological conditions of pH, temperature and time. These prebiotics included lactulose, different mixtures of commercial galactooligosaccharides, and an emerging prebiotic as novel galactooligosaccharides derived from lactulose (31). Recently, a pioneering approach, based on the study of the trans-β-galactosylation activity of the pig β-galactosidase embedded in the brush border membrane vesicles, has provided significant insights into the reaction mechanisms involved in the human digestion of dietary carbohydrates and has informed the development of non- or slowly-digestible carbohydrates (32). Lastly, a novel optimized *in vivo* and *in vitro* ileal fermentation assay, based on growing pigs as an animal model for simulating digestion in human adults, has been reported last year (33). Interestingly, the predicted values for ileal organic matter digestibility (calculated on the basis of the *in vivo*/*in vitro* ileal fermentation) were quite similar to the values measured *in vivo*, warranting the potential use of appropriate, and refined *in vitro* approaches using intestinal material from growing pigs to replace *in vivo* studies, in relation to ileal digestibility.

## The Need for Harmonization and Standardization of an *in vitro* Test Methodology for Assessing Digestibility of Dietary Carbohydrates

Despite recent advances in developing *in vitro* methods to assess carbohydrate digestibility, there is an obvious need to design a standardized batch gastrointestinal digestion method based on physiologically relevant conditions. By considering the state-of-art, it is clear that the mucosal disaccharidases embedded in the small intestinal brush border membrane vesicles must be considered in addition to α-amylases, since they play a key role in the digestion of carbohydrates and in the subsequent uptake of monosaccharides by the intestinal mucosa (Table 1). Additionally, the use of mammalian digestive enzymes should be prioritized over the use of microbial enzymes since the former better reflect the carbohydrase activities of enzymes of the human gastrointestinal tract. However, there are still important uncertainties that prevent the development of a standardized methodology in the short-term. These include (but not exclusively) the following aspects:

- A large variety of enzymes from different sources have been used to date, also differing in the assays used to determine the enzyme activity. This clearly impairs an appropriate characterization of the carbohydrases used as well as the comparison of outcomes from different studies.
- The regular supply of commercial pig mucosal small intestinal enzymes is nonexistent whilst that of rats is restricted.
- The expression of mucosal carbohydrases greatly varies among the different segments of the small intestine and is also driven by the type of diet, as well as the host genetics, health status and age, among other factors (34).

Possible solutions to overcome these drawbacks could be the production of individual recombinant mucosal carbohydrases that should provide an easier handling, as well as a more predictable and controllable enzyme activity (23). Likewise, estimations of the relative amount of the different mammalian mucosal carbohydrases have been already reported (35). These values could be useful to establish a physiologically relevant ratio among the different mucosal carbohydrases. Thus, the sucrase-isomaltase complex seems to be the major mucosal carbohydrase, followed by the maltase-glucoamylase complex, whereas the trehalase is the less abundant enzyme (36). Finally, a key aspect that could help to drive harmonization and allow more effective comparison of different *in vitro* digestibility conditions would be to include appropriate suits of control digestible carbohydrates (for instance, maltose, sucrose, starch-derived oligosaccharides, lactose, etc.) and well-recognized non-digestible comparator carbohydrates (such as lactulose or fructooligosaccharides).

Although there is still a considerable way to go in the full understanding of the physical and chemical dynamics of mammalian intestinal mucosal carbohydrases, the growing relevance, based on their beneficial impact of human health, of non-digestible carbohydrates emphasizes the necessity of developing a cost-efficient, fit-for-purpose, easily implemented, and physiologically relevant *in vitro* methodology which considers all human carbohydrases involved in dietary carbohydrate digestion.

## Author Contributions

All authors listed have made a substantial, direct and intellectual contribution to the work, and approved it for publication.

### Conflict of Interest Statement

The authors declare that the research was conducted in the absence of any commercial or financial relationships that could be construed as a potential conflict of interest.
